# Geosmin-producing Species of *Coelosphaerium* (Synechococcales, Cyanobacteria) in Lake Shinji, Japan

**DOI:** 10.1038/srep41928

**Published:** 2017-02-14

**Authors:** T. Godo, Y. Saki, Y. Nojiri, M. Tsujitani, S. Sugahara, S. Hayashi, H. Kamiya, S. Ohtani, Y. Seike

**Affiliations:** 1 Shimane Prefectural Institute of Public Health and Environmental Science, 582-1 Nishihamasada, Matsue, Shimane 690-0122, Japan; 2 The United Graduate School of Agricultural Sciences, Tottori University, 4-101 Koyama-cho minami, Tottori, Tottori 680-8553, Japan; 3 Graduate School of Science and Engineering, Shimane University, 1060 Nishikawatsu, Matsue, Shimane 690-8504, Japan; 4 Faculty of Life and Environmental Science, Shimane University, 1060 Nishikawatsu, Matsue, Shimane 690-8504, Japan; 5 Faculty of Education, Shimane University, 1060 Nishikawatsu, Matsue, Shimane 690-8504, Japan

## Abstract

In Lake Shinji, Japan, periodic outbreaks of musty odour have occurred since mid-May 2007. Although the substance responsible for the odour was identified as geosmin, the odour-producing organism was unknown. We cultivated an axenic unialgal strain and determined that a species of *Coelosphaerium* (Synechococcales) was responsible for the production of geosmin in Lake Shinji. Our analysis was conducted using gas chromatography/mass spectrometry to determine the odorous compound. To determine the algae species, it was observed by optical microscopy to describe its morphological characteristics and the polymerase chain reaction was used to characterise the nucleotide sequence of the 16S rRNA gene and the 16S-23S rRNA internal transcribed spacer region. In addition, we explored the relationship between the number of cells of the *Coelosphaerium* sp. and the concentration of geosmin. In conclusion, geosmin, the cause of the musty odour in Lake Shinji in autumn 2009, was produced by *Coelosphaerium* sp., and to our knowledge, this is the first report of a geosmin-producing species in the family Coelosphaeriaceae.

In aquatic ecosystems, an undesirable taste and odour results in a decline in the value of drinking water and fisheries. The majority of all biologically caused outbreaks of a distinctive taste and odour in drinking water and fisheries are caused by geosmin (E-1,10-dimethyl-E-9-decanol) and 2-MIB (2-methylisoborneol). The smell produced by these two substances is called musty odour. Musty odour is produced by certain species of cyanobacteria, actinomycetes, moulds, fungi, and myxobacteria^1^,^2^,^3^,^4^.

The first organisms that were confirmed to produce musty odour were actinomycetes. Safferman *et al*.^5^ detected geosmin in a culture of *Symploca muscorum* (cyanobacteria). Many studies have now reported that various cyanobacteria produce geosmin^6^,^7^,^8^,^9^. Geosmin was produced by some Oscillatoriales (e.g., *Phormidium*) and Nostocales (e.g., *Dolichospermum*). In Synechococcales, coccoid *Synechococcus* and some filamentous genera (e.g. *Leptolyngbya*) have been reported to produce geosmin; however, no geosmin-producing species have reported in the colonial genera of the family Coelosphaeriaceae^9^.

At Lake Shinji, in the eastern part of Shimane prefecture in Japan, an intense musty odour was noted at the lakeside in September 1993, affecting the bivalve fishery. However, no odorous substance or odour-producing organism was identified^10^. In mid-May 2007, many people around Lake Shinji smelled a musty odour and reported off-flavours in the shellfish *Corbicula japonica* and *Lateolabrax japonicus*. Geosmin was determined by the Shimane prefectural government to be the odorous compound responsible, which was detected using a solid phase gas chromatograph equipped with a quadrupole mass spectrometer. However, the causative organism was unknown, because microscopic examination of the lake water did not reveal any of the cyanobacteria known to be geosmin producers. A few actinomycetes producing geosmin were present, but the number of these organisms was insufficient to produce the amount of geosmin present.

In the following year (2008), musty odour was again noted at Lake Shinji. Godo *et al*.^11^ separated suspended substances from the lake water using a step density gradient. The number of cells of *Coelosphaerium kuetzingianum* correlated with the concentration of geosmin in the separated portions of water samples. These result suggested that *C. kuetzingianum* produced geosmin.

The scientific name *C. kuetzingianum* was used to refer to the specimens of *Coelosphaerium* in Lake Shinji during monthly monitoring of phytoplankton^12^ and in a study of geosmin-forming species of *Coelosphaerium*^11^. However, in the present study, we identified the organism only to the genus level because there are size differences in the colonies and the number of cells per colony between specimens from Europe and Lake Shinji (see taxonomic remarks in the Results section), suggesting that these may be different species.

Assuming that *Coelosphaerium* species produce geosmin, we cultivated an axenic strain of *Coelosphaerium* species. We identified it using an optical microscope and polymerase chain reaction (PCR) analysis, measured geosmin by gas chromatography mass spectrometry (GC/MS), and checked that the number of *Coelosphaerium* sp. correlated with the concentration of geosmin.

## Results

The species composition of phytoplankton in the surface layer of water was confirmed by Shimane Prefectural Institute of Public Health and Environmental Science. The sample contained cyanobacteria, including *Synechocystis* sp., *Synechococcus* sp., *Aphanocapsa* sp., *Merismopedia* sp., *Eucapsis* sp., and *Coelosphaerium* sp. Phytoplankton that are known to produce musty odour were not present. We suggested in our previous study that *Coelosphaerium* sp. produced the geosmin^11^. On the day of sample collection, musty odour was not noted at the field site, but after the sample was brought back to the laboratory, the odour was slightly noticeable. The total geosmin concentration of this sample was 84 ng L^−1^, and the dissolved geosmin concentration of this sample was 12 ng L^−1^.

### Identification and morphological characteristics of Coelosphaerium sp

**Figure 1:**
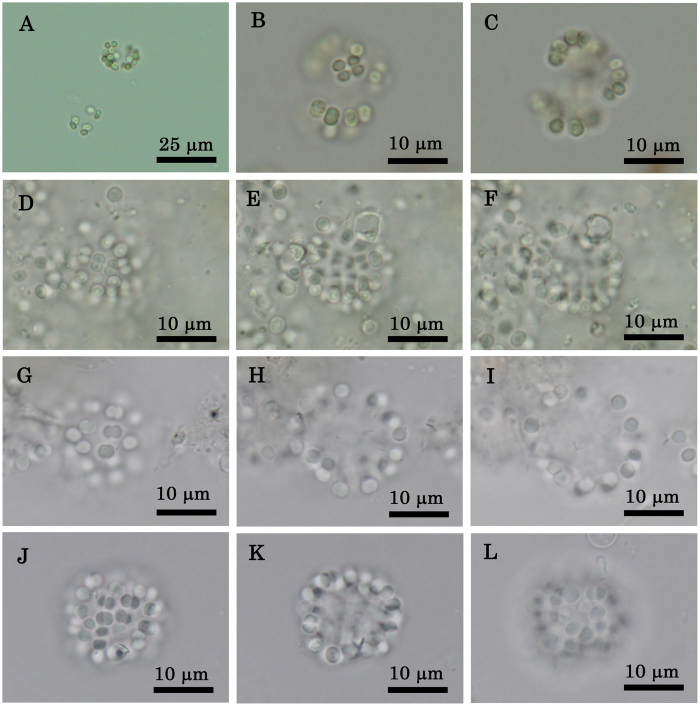
Morphological characteristics of *Coelosphaerium* sp. in the field samples in May 2007, 2008, and 2009. (**A**–**C**) Live colonies of *Coelosphaerium* sp. sampled in May 2007. (**A**) Colonies of different sizes. (**B**) Surface view of colony. (**C**) Optical section of colony. (**D**–**L**) Colonies preserved in a glutaraldehyde–formaldehyde solution. (**D**–**F**) Spherical colony sampled in May 2007. (**D**) Surface view of colony. (**E**) Optical section of colony. (**F**) Bottom view of colony. (**G**–**I**) Spherical colony sampled in May 2008. (**G**) Surface view of colony. (**H**) Optical section of colony. (**I**) Bottom view of colony. (**J**–**L**) Spherical colony sampled in May 2009. (**J**) Surface view of colony. (**K**) Optical section of colony. (**L**) Bottom view of colony.

The specimens that produced the musty odour from 2007 to 2009 in Lake Shinji were identified as a colonial-living species of the genus *Coelo*s*phaerium*. This species was dominant in the samples collected in May 2007 (no cell numbers data), May 2008 (1.50 × 10^7^ cells L^−1^), and May 2009 (1.06 × 10^7^ cells L^−1^). Morphological characteristics of *Coelosphaerium* sp. in the field samples are shown in Fig. 1 and Table 1.

Colonies were free-floating, spherical to widely oval in shape, and lacking a mucilaginous envelope. They ranged from 13 to 23 μm in length, 11 to 20 μm in width, and 9 to 23 μm in thickness. Colonies were sometimes divided into two subcolonies. The number of cells per colony ranged from 14 to 80. Cells were located on the periphery of each colony and lacked mucilaginous stalks. Cells were blue-green, spherical to hemispherical in shape, lacking aerotopes, 2–3 μm in diameter. During reproduction, cells utilized binary division to divide into two cells of equal size.

### Taxonomic remarks

The morphological characteristics that we observed coincide well with the descriptions of the genus *Coelosphaerium*^13^,^14^,^15^. The shape of *Coelosphaerium* colonies is similar to that of *Snowella* and *Coelomoron* within the family Coelosphaeriaceae^16^. Unlike *Snowella* sp., specimens in Lake Shinji had no mucilaginous stalks radiating from the colonial centre. Unlike *Coelomoron*, which has elongated cells, the cells in Lake Shinji were spherical to hemispherical. Thus, we identified the colonial specimens in Lake Shinji as a species of *Coelo*s*phaerium*.

Nägeli^13^ described *C .kuetzingianum* on the basis of a specimen collected from Zürich, Europe. The shape of the colony (spherical) and cell size (2.2 μm) of specimens in Nägeli’s original description^13^ correspond well with those of the organisms found in Lake Shinji. However, the diameter of the colony and the number of cells per colony that Nägeli reported differed greatly from the observed specimens in Lake Shinji. The diameter of the colony was up to 44 μm and the number of cells per colony was approximately 400, according to the original description. In contrast, the specimens in Lake Shinji were 13–23 μm in diameter and there were 14–80 cells per colony (Table 1). Komárek and Anagnostidis^15^ reported that the diameter of the colony of *C .kuetzingianum* in Europe was up to 100 μm. Tilden^17^ reported that colonies of this species were 30–90 μm in diameter in the United States. European and North American specimens appear to have larger-diameter colonies and many more cells per colony than do the specimens of Lake Shinji.

We used the scientific name *C .kuetzingianum* to refer to the specimens of *Coelosphaerium* in Lake Shinji during the monthly monitoring surveys of phytoplankton^12^ and during the study of geosmin-forming species of *Coelosphaerium*^11^. However, considering the differences in morphological characteristics mentioned above, we will no longer use *C .kuetzingianum* to refer to these organisms and will limit the identification to the genus level in the present study.

### Morphological characteristics of field specimens and strain G2

**Figure 2:**
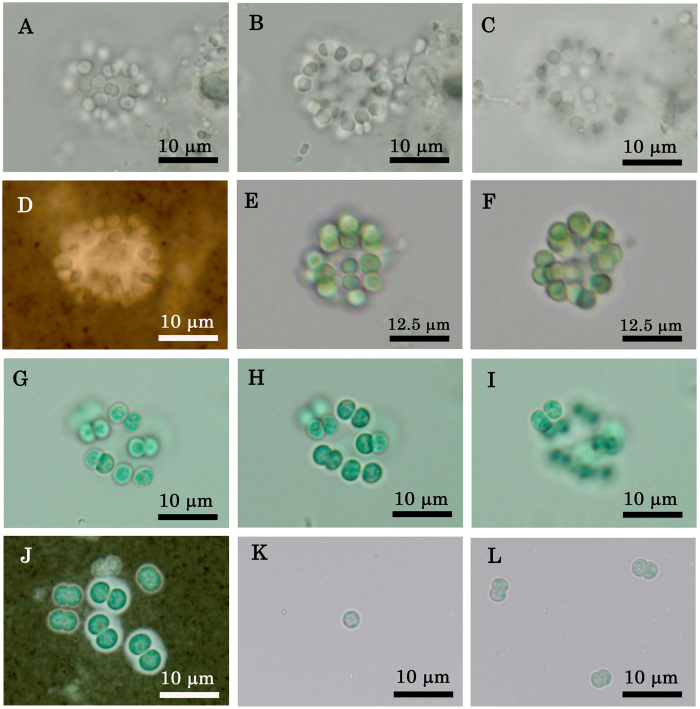
Morphological characteristics of *Coelosphaerium* sp. isolated in October 2009 from Lake Shinji and colonies or cells of strain G2. (**A**–**D**) Oval colony sampled in October 2009, preserved in a glutaraldehyde–formaldehyde solution. (**A**) Surface view of colony. (**B**) Optical section of colony. (**C**) Bottom view of colony. (**D**) Mucilage was not observed around the colony after negative staining with India ink. (**E**–**L**) Strain G2 in culture. (**E**–**F**) Oval colony after one month of isolation in M11 medium. (**E**) Surface view of colony. (**F**) Optical section of colony. (**G**–**J**) Colony composed of 16 cells of strain G2 in 1/10IMK medium. (**G**) Surface view of colony. (**H**) Optical section of colony. (**I**) Bottom view of colony. (**J**) Cells with narrow mucilage revealed by negative staining with India ink. (**K**–**L**) One or two cells in CA medium.

In October 2009, the musty odour was noted at Lake Shinji. We isolated a strain of *Coelosphaerium* sp. (strain G2) from a sample of surface water. Morphological characteristics of these field specimens are shown in Table 1. A colony of *Coelosphaerium* sp. sampled in October 2009 is shown in Fig. 2A–D.

Strain G2 first formed colonies after isolation for one month in M11 medium (Fig. 2E and F and Table 2). The photograph shows one colony, after one month. The colony was widely oval in shape, 20 μm in length, and 18 μm in width. The number of cells per colony was about 20. Cells were blue green, spherical, lacking aerotopes and 4.0–5.0 μm in diameter. Cells divided into two cells of equal size (binary division). Cells in the M11 medium were somewhat larger than those in the field samples (2–3 μm).

Colonies divided into two or four cells in M11 medium after two months of isolation. Because strain G2 showed unstable growth (i.e. continuous cultivation of strain G2 resulted in pale cultures that was slower growth rates) in M11 medium, we moved the strain to CA medium. Although CA medium promoted the growth of strain G2, colonies did not form and the organism remained unicellular or existed as two cells from 2009 to 2014 (Fig. 2K and L). In July 2015, after six years of isolation, the strain had developed a few colonies containing 16 cells, with cells loosely arranged at the periphery of the colony (Fig. 2G–I). During this time, strain G2 was cultured in 1/10 IMK medium containing 10% sea water at 20 °C, in a 12:12 light and dark cycle. However, the strain was primarily unicellular or present as two cells. The culture conditions that would generate a colony of strain G2 are unknown.

### Axenic nature of cultivated strain

We proved the axenic state of the culture based on the following three criteria: (1) The axenic check medium on which the cultivated strain G2 was inoculated was clear. (2) A test by staining with ethidium bromide revealed that only the red point of the *Coelosphaerium* sp. (spherical and 2–3 μm of diameter) was observed. (3) Although some bands were detected in 16S rRNA PCR-DGGE analysis, the sequences of the bands matched each other and the partial sequence of *Coelosphaerium* sp. G2. From these results, we concluded that the strain G2 was an axenic cyanobacterial culture.

### Analysis of nucleotide sequence of the 16S rRNA gene and the 16S-23S rRNA ITS region

A 2028-bp sequence was determined from a cell of strain G2, which included the 16S rRNA gene and the 16S-23S rRNA internal transcribed spacers (ITS) region. BLAST searches revealed that the most closely related nucleotide sequences were from uncultured *Coelosphaerium* spp. (EF638722 and EF638723)^18^. They were detected in Okawa Bay in New Zealand by cultivation-independent techniques. The sequence also exhibited high levels of sequence identity (98%–99% and 93%, respectively) with 16S rRNA genes of *Snowella litoralis* (AJ781041, AJ781039, and AJ781042)^19^ and *Coelomoron pusillum* AICB1012 (KJ746507).

**Figure 3:**
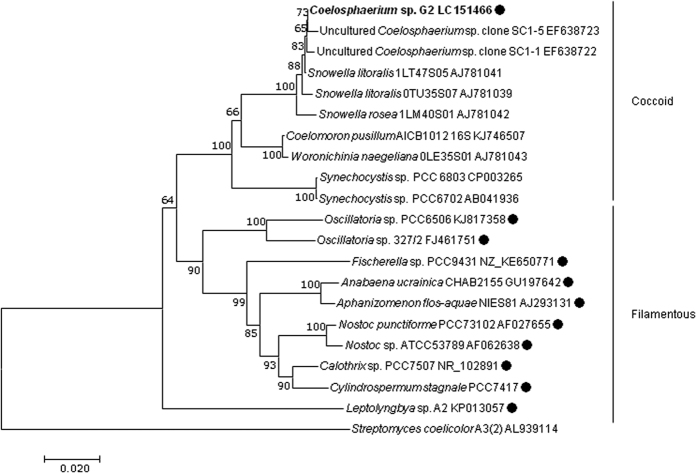
Phylogenetic analysis. Phylogenetic tree of *Coelosphaerium* sp. G2 and the related cyanobacterial group based on 16S rRNA gene sequences (1076 bp). The phylogenetic tree was constructed using the neighbour joining method and 1000 bootstrap replicates, constructed with MEGA7 software program. Bootstrap values above 60% are shown at the nodes. The accession numbers are shown to the right of the strain name. Sequences from *Coelosphaerium* sp. G2 are indicated in bold. Closed circles indicate geosmin producers of cyanobacteria. The cell form is indicated to the right of the figure. The scale bar indicates substitutions per site. The actinomycete *Streptomyces coelicolor* A3(2) was used as an outgroup.

The phylogenetic trees generated for the partial 16S rRNA gene sequence (1076 bp) are shown in Fig. 3. In the phylogenetic tree, strain G2 formed a branch with uncultured *Coelosphaerium* spp. and *Snowella* spp., and was grouped in a coccoid clade with other coccoid cyanobacterial strains, including *Synechocystis* spp. Strain G2 of Coelosphaerium sp. is the first known strain that produces geosmin in the family Coelosphaeriaceae.

### Identification of odorous substance

**Figure 4:**
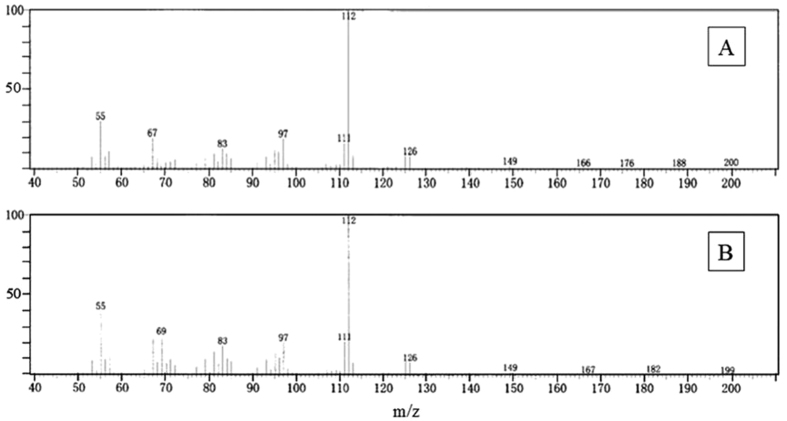
Mass spectrometry analysis. Mass spectrum of (**A**) a peak in the geosmin standard solution and (**B**) a peak in strain G2 that appears to be geosmin.

A peak of the mass chromatogram resulting from strain G2 showed the same retention time as a peak of geosmin in the mix standard solution. Another peak of strain G2 did not show the same retention time as 2-MIB. Mass spectrometry of a geosmin peak in standard solution and the peak in strain G2, which appears to be geosmin, is shown in Fig. 4. Both mass spectra are very similar. The odorous substance produced from cultivated stock was identified as geosmin.

### Relationship between the number of cells of Coelosphaerium sp. and the concentration of geosmin

No *Coelosphaerium* sp. were observed on 16 and 18 September, 2009. The number of cells of *Coelosphaerium* sp. increased from 4.8 × 10^5^ cells L^−1^ on September 24 to the maximum value of 6.12 × 10^7^ cells L^−1^ on October 27. The number of cells declined to 4.64 × 10^7^ cells L^−1^ by November 4.

The geosmin concentration increased in raw and filtered water from 3 ng L^−1^ and 1 ng L^−1^, respectively, on September 16. On September 24, the concentrations of geosmin were 12 ng L^−1^ and 4 ng L^−1^, respectively. On October 27, the geosmin concentration in raw and filtered water were detected at maximum values of 640 ng L^−1^ and 20 ng L^−1^, respectively. The respective concentrations then decreased to 413 ng L^−1^ and 17 ng L^−1^ on November 4. The change in the number of cells of *Coelosphaerium* sp. paralleled that of the geosmin concentration. The musty odour was not smelled until October 20. Meanwhile, in the laboratory, a slight musty odour was detected from a sample taken after October 5.

## Discussion

### Geosmin-producing Coelosphaerium sp

We cultivated the strain G2 from the surface water of Lake Shinji. Morphological characteristics and PCR analysis confirmed that strain G2 contained only *Coelosphaerium* species. Strain G2 was shown to be an axenic strain through multiple checks, namely axenic medium, staining with ethidium bromide, and 16S rRNA PCR-DGGE analysis. Mass spectrometry proved that strain G2 produced geosmin, linking its production for the first time to a *Coelosphaerium* species.

According to Persson^6^, in order to establish whether an organism produces odours, the following criteria should be used:

1. Ecological evidence: concurrence of odour and organism in the field.
2. Isolation of organism and proof of odour production in the laboratory (i.e., sensory characterization of cultures, confirmation of odour production, and description of odour).
3. Chemical identification and sensory characterization of odour compounds.

**Figure 5:**
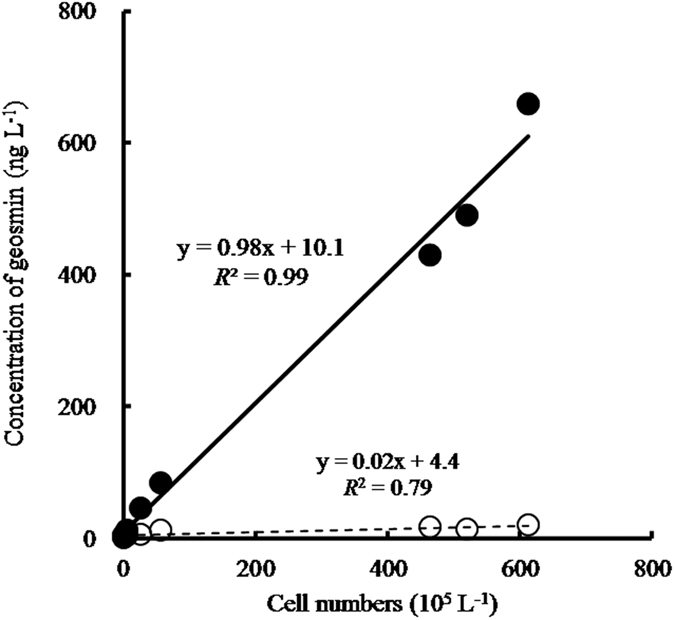
Correlation analysis. Correlation plots between the concentration of geosmin and cell numbers of *Coelosphaerium* sp. from September 16 to November 4, 2009. Filled and open circles indicate raw and filtered sample, respectively.

Our analysis met these three criteria. In addition, we showed that a good correlation exists between the number of cells of *Coelosphaerium* sp. and the concentration of geosmin in the field (Fig. 5). Therefore, the cause of musty odour (geosmin) in Lake Shinji in autumn 2009 was *Coelosphaerium* sp.

Many cyanobacteria are known to produce geosmin and/or 2-MIB; however, Coelosphaeriaceae species were not known to be included among these geosmin-producing cyanobacteria^9^.

This is the first report of Coelosphaeriaceae species producing geosmin. In addition, it is the first report of an analysis of the nucleotide sequence of the 16S rRNA gene and the 16S-23S rRNA ITS region of this organism.

The threshold for the concentration of geosmin in the water that produces an odour is said to be 4–10 ng L^−1^ 7,20,21,22,23. In this investigation, concentration of total geosmin was 12 ng L^−1^ on September 24, 27 ng L^−1^ on September 30 and the after a while, value was higher, the musty odour was felt at a field or a laboratory after October 5. Concentration of dissolved geosmin was 12 ng L^−1^ on October 5 and the after a while, value was higher. These results suggest that smell of geosmin was associated with the dissolved in the water rather than the total geosmin (including algal cell). Godo *et al*.^24^ reported similar phenomena in Lake Shinji in Spring 2008.

At Lake Shinji, the presence of *Coelosphaerium* sp. is common in spring and fall when the water temperature is approximately 15 °C. We continue the monthly sampling of phytoplankton that began in 1969^12^. During this time, from 1969 to 2012, musty odour was recorded at Lake Shinji only five times. One of these times was based on a newspaper article in 1987 that was not associated with a scientific investigation, so it contains few details. In September 1993, strong musty odour was observed again at Lake Shinji. At that time, we were unable to measure the geosmin using GC/MS, but musty odour was assumed as geosmin. Here, no odour-producing organism was identified, but *Coelosphaerium sp.* was observed in the lake^10^. From November 1993 to March 1994, the number of cells of *Coelosphaerium sp.* increased and the species became the dominant plankton species, but no musty odour was noted. Starting in May 2007 and continuing until 2009, *Coelosphaerium sp.* was observed and musty odour was detected. After 2010, *Coelosphaerium sp.* was observed but there was no musty odour.

In Lake Shinji, *Coelosphaerium* sp. is a common species, but musty odour occurs only once every few years. Schrader *et al*.^25^ stated that even when *Coelosphaerium* sp. was observed in great abundance, no geosmin was detected in the same water sample. Oikawa and Ishitobi^26^ reported that *Phormidium tenue* was divided into three groups genetically in Lake Kamafusa in Japan, one of which did not produce a substance with musty odour. We propose that *Coelosphaerium* sp. also exist in two or more genetic groups in Lake Shinji, some of which produce geosmin. The gene associated with the production of geosmin in cyanobacteria has been identified^27^,^28^. Therefore, future research should identify in which species of *Coelosphaerium* this gene is present.

## Materials and Methods

### Study site

**Figure 6:**
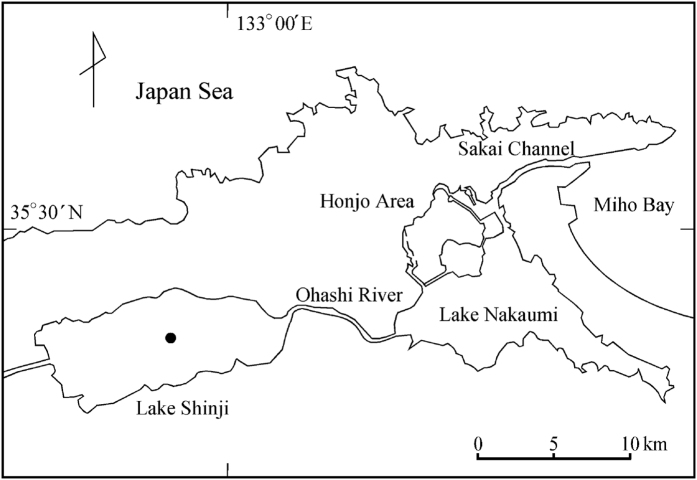
Study location. Lake Shinji, the surrounding area, and sampling location. Software: canvas, ver. x, URL: http://www.canvasgfx.com/en/legal-notices.

Lake Shinji (area 79.8 km^2^, mean depth 4.5 m) is a brackish lake in the eastern part of Shimane prefecture, Japan (Fig. 6). It mainly receives fresh water from the Hii River, which drains 70% of the lake’s drainage basin. An intermittent supply of highly saline water from Lake Nakaumi flows through Ohashi River into Lake Shinji. The degree of salinity is affected by rainfall and other factors^29^,^30^. Average salinity, from 1993 to 2012, was 3.5 PSU (Practical Salinity Unit) and varied from year to year. Average retention time, from 1993 to 2011, was 59.4 days considering the high-salinity water reversed from Lake Nakaumi through Ohashi River to Lake Shinji^31^. Highly saline water from Lake Nakaumi flowed into Lake Shinji as a dense current and formed a layer of water on the bottom of the lake^32^. During the summer, this layer readily became oxygen depleted and the concentration of nutrients under the halocline increased because of the high temperature and bioactivity^33^.

A monthly survey was conducted starting in 1969 to measure water quality factors and the species composition of phytoplankton. The number of members of dominant species and the relative frequency of the dominant species were recorded. *Coelosphaerium* sp. were consistently present in Lake Shinji between 1969 and 2012, except for in 2005^12^.

### Field survey

A weekly survey was performed at a central point of Lake Shinji (35.4504N, 132.9599E) from September 16 to November 4, 2009. Water samples 2 L in volume were collected by a stainless steel bucket and were transported to a laboratory within 4 hours. Immediately, total concentrations of geosmin in the water were measured and the number of cells of *Coelosphaerium* sp. were counted.

In the field, water temperature, electric conductivity, and dissolved oxygen were measured from the surface to the bottom at 1 m intervals using a sensor (HACH Hydrolab MS5 Denver).

### Establishment of laboratory culture

Surface water samples 20 L in volume were collected by bucket at a central point of Lake Shinji on 5 October 2009, a day on which musty odour was noted. The samples were transported to a laboratory within 4 hours, preserved in a refrigerator at 4 °C, and used to start cultivation within a week.

Single-colony isolation was performed using the pipette-washing method^34^ under a stereo microscope. The strain G2 of *Coelosphaerium* sp. was maintained in test tubes containing 10 mL of M-11 medium^35^ under the following growth conditions: temperature 20 °C, light approximately 25 μmol m^−2^ s^−1^ (provided by cool white fluorescent tubes), and 12:12 light and dark cycles. As the growth of strain G2 was unstable in M11 medium, we used CA^36^ and IMK (salinity 3 PSU) media as alternatives.

### Observations of morphological characteristics and counting of cell numbers

The field sample was condensed 100-fold as follows. Raw sample water (198 mL) was filtered through a 0.45 μm membrane filter (Millipore Corp.) under low vacuum conditions. The deposits were carefully scraped from the filter using a small spatula and diluted to 2 mL of sample water. Following this, 2 mL of 2.5% glutaraldehyde was added to the 100-fold condensed sample. Once the sample had settled, the deposits were removed and resuspended in 2 mL of 5% formalin. This process means that the sample was concentrated 100-fold.

The shapes of colonies and cells were observed under an optical microscope with a fluorescent attachment (Olympus BX60, Japan) at a magnification of 400x or 1000x. To measure the size of colonies and cells, representative groups of more than 10 colonies from each sample were measured at random with a micrometre at a magnification of 1000x.

The number of cells in a 1.0 × 10^−7^ L volume of the 100-fold condensed sample was counted using a Thomas haemocytometer in a 1-mm^2^ field under an optical magnification of 400x. This measurement was repeated three times, and the mean values were used to estimate the number of cells.

### Analysis of nucleotide sequences

DNA was extracted from the cells in strain G2 using ISOPLANT (Nippon Gene Co., Ltd., Toyama, Japan), according to the manufacturer’s instructions. The approximately 2-kb fragment, including the 16S rRNA gene and the 16S-23S rRNA ITS region, was amplified using KOD plus polymerase (Toyobo Co., Ltd., Osaka, Japan) with fD1 (5′-AGAGTTTGATCCTGGCTCAG-3′)^37^ and 23S-R130-16 (5′-GGGTTTCCCCATTCGG-3′). The PCR amplification mixture was prepared according to the manufacturer’s instructions, and the PCR cycle was composed of a pre-run at 94 °C for 2 min, 30 cycles of denaturation at 94 °C for 15 sec, annealing at 50 °C for 30 sec, and extension at 68 °C for 2.5 min, followed by a final extension at 68 °C for 10 min. The nucleotide sequence was determined using a BigDye Terminator Cycle Sequencing Ready Reaction Kit and an ABI PRISM 3130xl Genetic Analyzer (Applied Biosystems, CA, USA) as described previously^38^. The primers used were fD1, F338 (5′-ACTCCTACGGGAGGCAGCAG-3′)^38^, 926 F (5′-AAACTCAAAGGAATTGACGG-3′)^39^, 1512 f (5′-GTCGTAACAAGGTAGCCGT-3′)^40^, 518r (5′-ATTACCGCGGCTGCTGG-3′)^38^, 907r (5′-CCGTCAATTCCTTTGAGTTT-3′)^41^, rP2 (5′-ACGGCTACCTTGTTACGACTT-3′)^37^, and 23S-R130-16. The sequence was compared with those in the DNA Data Bank of Japan (DDBJ) using the BLAST algorithm (http://www.ddbj.nig.ac.jp/searches-j.html). The phylogenetic tree was constructed using the neighbour joining method with the MEGA7 software program^42^. The nucleotide sequence determined in this study has been deposited in the DDBJ, European Nucleotide Archive (EMBL), and GenBank databases under accession number LC151466.

### Confirmation of axenic strain

We used three methods to confirm that strain G2 was axenic. First, strain G2 was incubated for three weeks using six different axenic check mediums: B-I, B-II, B-III, B-IV, B-V^43^, and YT^44^. Secondly, to verify that there were no bacterial cells present, samples of strain G2 were observed under a microscope after staining with ethidium bromide^45^,^46^. Finally, to confirm that no extra band from contaminating bacteria was present, 16S rRNA PCR-denaturing gradient gel electrophoresis (DGGE) was performed as described previously^38^,^41^. Briefly, the 16S rRNA fragment was amplified from DNA extracted from strain G2 with the primers F338GC (5′-CGCCCGGGGCGCGCCCCGGGCGGGGCGGGGGCACGGGGGGACGGGGGGACTCCTACGGGAGGCAGCAG-3′)^47^ and 907r. The denaturing gradient gel was prepared as 6% polyacrylamide with 30 to 60% denaturing gradient. Electrophoresis was performed at 100 V for 16 hours at 60 °C. The consistency shared identity of the nucleotide sequence between the excised bands and *Coelosphaerium sp.* G2 was confirmed by nucleotide sequencing.

### Analysis of geosmin

Geosmin was analysed using headspace GC/MS. The measurements were conducted using a headspace autosampler (TurboMatrix 40, Perkin Elmer, USA) and gas chromatograph (GC-2010, Shimadzu, Japan) equipped with a quadrupole mass spectrometer (QP2010 Plus, Shimadzu, Japan). Geosmin standard solution and deuterated internal standard of geosmin (d_3_-geosmin) were purchased from Wako Chemicals (Japan). NaCl was heated for over 4 hours at 450 °C. The reagents used were not contaminated prior with geosmin.

A 10-mL sample, to which 3.5 g NaCl was added, was placed into the vial tube with deuterated internal standard of geosmin (d_3_-geosmin) and sealed with an aluminium stopper. After the sample was preheated at 80 °C for 30 min, the gas phase in the tube was introduced into the capillary column of GC/MS. The GC column (Rtx-5MS, 30 m by 0.25 mm by 1.0 μm film thickness, Restek, USA) was held at 35 °C for 1 min, then increased by 30 °C/min to 150 °C, then increased by 5 °C/min to 200 °C, and then increased by 30 °C/min to 230 °C, and the final temperature was held for 5 min at 100 kPa of helium (He) gas. GC/MS analysis was carried out in scan mode (qualitative analysis) and in selected ion monitoring mode (m/z 112) (quantitative analysis). The range of the standard curve was from 1 ng L^−1^ to 100 ng L^−1^. For each sample, the analysis was performed twice. If the analytical value surpassed the maximum limit, we reanalysed a sample diluted with water to a level required to achieve sample concentration in the appropriate range.

## Additional Information

**How to cite this article**: Godo, T. *et al*. Geosmin-producing species of *Coelosphaerium* (Synechococcales, Cyanobacteria) in Lake Shinji, Japan. *Sci. Rep.* **7**, 41928; doi: 10.1038/srep41928 (2017).

**Publisher's note:** Springer Nature remains neutral with regard to jurisdictional claims in published maps and institutional affiliations.

## Figures and Tables

**Table 1:** Morphological characteristics of *Coelosphaerium* sp. collected from Lake Shinji when the musty odour was present, from 2007 to 2009.

Collecting date	Colony shape	Number of cells per colony	Length of colony (μm)	Width of colony (μm)	Thickness of colony (μm)	Cell shape	Diameter of cells (μm)
May 2007	Spherical to widely oval	14–40	13–19	11–15	13–20	Spherical to hemispherical	2.5
May 2008	Spherical to widely oval	19–80	14–20	12–20	9–23	Spherical to hemispherical	2.0–3.0
May 2009	Spherical to widely oval	21–75	13–17	13–17	13–19	Spherical to hemispherical	2.5–3.0
Oct. 2009	Spherical to widely oval	31–80	15–20	13–17	10–19	Spherical to hemispherical	2.5–3.0

**Table 2:** Morphological characteristics of strain G2, isolated from Lake Shinji.

Date of observation	Shape of Colony	Number of cells	Length of colony (μm)	Width of colony (μm)	Cell shape	Diameter of cells (μm)	Medium
2 Nov. 2009	Oval	ca.20	20	18	Spherical	4.0–5.0	M11
22 Dec. 2009	Spherical	2–4 (12)	16	14	Spherical to hemispherical	3.5–4.5	M11
23 July 2015	Oval	1–4 (16)	18	13	Spherical to hemispherical	3.5–4.0	1/10IMK
24 Mar. 2016	—	1–2	—	—	Spherical to hemispherical	2.5–3.0	CA

—: Colonies were not formed. (): Rarely observed
